# An efficient convolutional neural network-based diagnosis system for citrus fruit diseases

**DOI:** 10.3389/fgene.2023.1253934

**Published:** 2023-08-24

**Authors:** Zhangcai Huang, Xiaoxiao Jiang, Shaodong Huang, Sheng Qin, Su Yang

**Affiliations:** ^1^ Guangxi Key Laboratory of Brain-Inspired Computing and Intelligent Chips, School of Electronic and Information Engineering, Guangxi Normal University, Guilin, China; ^2^ Department of Computer Science, Swansea University, Swansea, United Kingdom

**Keywords:** identification and quantification, high-latitude features, EfficientNetv2, VGG, U-net

## Abstract

**Introduction:** Fruit diseases have a serious impact on fruit production, causing a significant drop in economic returns from agricultural products. Due to its excellent performance, deep learning is widely used for disease identification and severity diagnosis of crops. This paper focuses on leveraging the high-latitude feature extraction capability of deep convolutional neural networks to improve classification performance.

**Methods:** The proposed neural network is formed by combining the Inception module with the current state-of-the-art EfficientNetV2 for better multi-scale feature extraction and disease identification of citrus fruits. The VGG is used to replace the U-Net backbone to enhance the segmentation performance of the network.

**Results:** Compared to existing networks, the proposed method achieved recognition accuracy of over 95%. In addition, the accuracies of the segmentation models were compared. VGG-U-Net, a network generated by replacing the backbone of U-Net with VGG, is found to have the best segmentation performance with an accuracy of 87.66%. This method is most suitable for diagnosing the severity level of citrus fruit diseases. In the meantime, transfer learning is applied to improve the training cycle of the network model, both in the detection and severity diagnosis phases of the disease.

**Discussion:** The results of the comparison experiments reveal that the proposed method is effective in identifying and diagnosing the severity of citrus fruit diseases identification.

## 1 Introduction

Citrus is cultivated worldwide for its high commercial and nutritional value (Yang et al., 2022). The popularity of citrus cultivation in southern China has endured because of its good economic returns, especially in Guangxi (Zhou, 2020). With the great growth of citrus cultivation, the direct economic losses caused by citrus diseases are also climbed. Citrus canker is one of the major diseases affecting the quality of citrus, which is difficult to eradicate (Conti et al., 2020). Therefore, prompt treatment of citrus diseases is particularly important. To achieve this, the accurate identification of the disease type in citrus fruit and the accurate assessment of its severity is indispensable.

Many techniques are used for the identification of citrus diseases. Computer vision is one of the important methods, which is divided into pre-processing, segmentation, feature extraction and final classification. Pre-processing refers to the optimization of plant images to prepare for the next image processing. Common methods include image binarisation, noise reduction, enhancement, geometric changes and interpolation. Segmentation extracts feature such as size, colour and texture from an image by dividing it into different regions. Finally, the image is classified based on the extracted feature information (Lee et al., 2017). The refinement of accurate diagnostic computer systems has solved many of the problems of plant disease identification. Enables computer vision technology to be used in a wide range of practical scenarios. However, traditional computer vision techniques rely on manual feature extraction algorithms, which require high-quality data sets, making it difficult to achieve the accuracy expect from detection.

The progress of machine learning algorithms promotes the emergence of new methods. Deep Learning (DL) is regarded as the most potential and future computing technology in modern agriculture because of its high accuracy in classification and recognition tasks. Hence, DL plays an indispensable role in the automation of disease identification and detection. Especially, due to the excellent performance of Convolutional Neural Networks (CNNs) in image feature extraction, it is widely used for fruit recognition and prediction by providing an automatic feature extraction scheme without human intervention (Vasconez et al., 2020). CNN automatically extracts relevant features through training a large number of data sets, thus eliminating the traditional manual feature extraction link. The accuracy of the former is often much higher than that of the latter.

Different CNN models show different efficiencies. Depending on the database in question, the CNN with optimized depth, width and resolution may lead to much-improved results. Improving the network from these three aspects also means improving the network performance, and strengthening feature extraction capabilities. Features are divided into basic features and high-dimensional features, and network improvements nowadays tend to focus on the former rather than the latter. This results in models that increased complexity but struggle to reap corresponding performance gains. Improvements in high-latitude feature extraction are gaining attention in models saturated with basic feature extraction. High-latitude features are features that are extracted from multiple scales to exploit the multi-scale information of the image, in a way that is not limited to only one dimension. The enhanced capability of high-latitude feature extraction can improve the generalization performance of the network model and enhance the adaptability of the model to different datasets. Therefore, it is challenging to improve the ability of the model to extract high-dimensional features. and the quality of the extracted high-dimensional features can be judged by the accuracy of the disease identification. Once the type of disease has been accurately determined, the next challenge is to quantify the severity of the disease to determine the dose of medication.

Indeed, it is difficult to gauge the effectiveness of management practices without a quantifiable measure. Traditionally, the human eye relies on experience with the help of measurement aids to determine the severity of the disease, but this method lacks accuracy (Hassan et al., 2021). On the other hand, the traditional machine learning methods cannot quantify the severity, they can only determine the interval of the disease, such as early or late stages. It cannot give good advice on the progression and subtle changes in some subsequent diseases because it cannot achieve sufficient detection accuracy (Wang et al., 2017). Benefiting from the extension of the DL model, the image can be detected with sufficient resolution. Through pixel-by-pixel detection, the highest accuracy can be achieved when calculating the severity of the disease. However, the training of DL model depends on a large number of tagged image data, Therefore, collecting data for training is one of the challenges for disease severity analysis.

The main contributions of this study are: 1) A citrus disease detection system has been constructed, which is divided into a phase for the identification of disease species and a phase for the diagnosis of their severity. 2) A fast and accurate model for citrus fruit disease diagnosis is constructed by integrating InceptionV1 and EfficientNetV2. 3) In the system proposed in this paper, Transfer Learning (TL) is used to import the initialization weights of the network to reduce the training cycle of the model and compare it with the advanced models of disease diagnosis. 4) Estimation models are constructed to quantify the severity of citrus diseases, and the performance of different segmentation models is compared on our dataset.

The rest of the paper is organized as follows: Section 2 introduces the background of disease identification and severity quantification. Section 3 outlines the materials and methods of the proposed models for disease identification, and the experiment results using other advanced DL models are discussed and compared with the proposed method. The architecture and implementation of disease severity analysis are described in Section 4. Finally, the contribution of this study and plans are summarized in Section 5.

## 2 Related work

This section reviews the methods of disease recognition and semantic segmentation. Representative methods applied to these two fields in engineering are presented.

### 2.1 Disease recognition

Traditional manual methods of identifying citrus diseases often make identification inefficient and difficult to achieve the desired level of accuracy due to the tedious nature of the identification process and the variability of the preceding and following processes (Ismail and Malik, 2021). Compared with traditional manual recognition, computer vision-based technology can provide better solutions for citrus disease recognition. Images contain many visible features including textures, shapes, and colors. The machine learning methods extract the feature information contained in the image through the algorithm processing of the citrus image, to achieve the purpose of citrus classification. Citrus images are detected by a multispectral imaging sensor. Moreover, an approach for citrus classification using threshold processing is proposed in (Abdelsalam and Sayed, 2016). Adaptive neuro-fuzzy inference systems and linear, and nonlinear regression methods are used to grade citrus fruits (Sabzi et al., 2017). A system for classifying diseases of orange using multiclass Support Vector Machines (SVM) and calculating the severity of diseases using fuzzy logic is proposed in (Behera et al., 2018). The automatic citrus grading detection is performed by using BP neural network (Chen et al., 2018). These methods are interpretable and have the features of a high correct recognition rate compared with the manual method but the tediousness of the feature extraction process and the loss of features due to dimensionality reduction are challenges that need to be addressed (Chao et al., 2021). Although these methods are a significant improvement over manual methods, the non-automatic nature of feature extraction in the recognition process has prevented their widespread use in practical production.

DL has been widely researched for its automated feature extraction process, and it can effectively reduce the loss of information caused by manual feature extraction algorithms. In particular, the rise of CNNs has raised enthusiasm for DL to a whole new level. VGG (Simonyan and Zisserman, 2014), AlexNet (Iandola et al., 2016) and GoogleNet (Szegedy et al., 2015) are classic representations of CNN models, although these models cannot achieve very high accuracy, they are still widely employed in the field of agricultural engineering. These networks only require an input image to actively extract the feature information embedded in the image, but the performance of their output fluctuates with the merit of the dataset. This means that these models cannot be applied in some complex environments, and the reason for this is the inadequate feature extraction capability of these network models. In recent years, based on the developed computer hardware, people begin to optimize the depth learning model from depth, width and resolution. So deep Residual Neural Networks (ResNet) (Wu et al., 2019), Xception (Avery et al., 2014), EfficientNet (Tan and Le, 2019) and other more generalized DL models emerged at the times required.

ResNet classifiers are trained to detect the defects of tomato fruit (da Costa et al., 2020) and achieved an average precision of 94.6%. By combining TL with ShuffleNet, a lightweight model (Context Driven Detection Network) is constructed to detect and classify surface defects in carrots (Deng et al., 2021). Achieving 99.82% and 93.01% accuracy in binary and multiclass classification, respectively. The conclusion that CNNs are more accurate than SVM is proved in (Fan et al., 2020) by comparing the performance of CNN and SVM in Apple defect detection. Three learning models: AlexNet, GoogleNet and ResNet50 are used to grade Okra (Raikar et al., 2020). The accuracies obtained are 63.45% for AlexNet, 68.99% for the GoogleNet model and 99% for ResNet50 which is better than the others. By training, testing and comparing ResNet, DenseNet, MobileNetV2, NASNet and EfficientNet, EfficientNet is proven to be the best fruit grading model (Ismail and Malik, 2021). The accuracy exceeded 98% on both the apple and banana datasets. Although these models have better accuracy than those classical models, it can be observed that these models lack the ability to extract multi-scale features. The lack of high latitude feature extraction capability makes the DL model unable to achieve ideal results on some similar data sets. At the same time, the optimization of depth, width or resolution means the increase of model parameters and the occupation of computing resources. Therefore, it is a challenge for all DL models to improve the high latitude feature extraction capability of the model and reduce computing resources.

### 2.2 Disease severity diagnosis

To effectively control and treat plant diseases, an accurate diagnosis of the severity of the disease is an integral part of the effective identification of the plant disease. Disease severity diagnosis can be used to improve crop yields and reduce the economic losses caused by plant diseases. Disease detection models at this stage are not suitable for disease severity diagnosis. More often, segmentation models are used to distinguish between diseased and healthy areas for the next step of disease severity analysis.

A fuzzy logic inference system based on the DeeplabV3+ model is proposed in (Ji and Wu, 2022) for automated detection and disease analysis of grapevine black measles disease. DeeplabV3+ is used to separate the infected and healthy areas and a fuzzy inference system is introduced to diagnose the disease severity. The method has been shown to have high classification accuracy and also to be able to accurately measure the severity of grapevine black measles disease under controlled conditions. Also based on DeeplabV3+, a DeeplabV3+ model with multi-scale inputs is proposed to improve image recognition and segmentation performance of cancerous areas in pathological sections of gastric cancer (Wang and Liu, 2021). By incorporating a unique nested jumping device in U-Net to generate semantically similar feature maps in the connected section, a model called U-Net++ is proposed (Cheng et al., 2018). By comparing the segmentation performance of a set of the most representative models (Deeplabv3+, U-Net and U-Net++) for the bull’s-eye region in ultrasound images. It is concluded that U-Net++ has the best performance compared to the other models, achieving a segmentation accuracy of more than 97% (de Melo et al., 2022). A real-time detection system for apple leaf disease detection is proposed in (Khan et al., 2022). The system divides the detection phase into 2 stages, initial and detection. The initial phase is used to differentiate between diseased and disease-free leaves, while the detection phase is used to detect disease-susceptible areas of the leaves. By combining VGG and U-Net, a system for diagnosing the severity of tomato leaf diseases is proposed in (Wspanialy and Moussa, 2020), obtaining results comparable to human assessments. The proportion of regions suggested as the most appropriate indicators of disease severity for plant diseases caused by fungi or bacteria, the ordinal classification is more applicable to diseases caused by viruses or insects.

## 3 Methodology

EfficientNetV2 (Tan and Le, 2021) and the Inception module are introduced in detail in this section and an improved EfficientNetV2 is applied to disease detection in citrus fruits. In addition to this, several different segmentation algorithms are compared to find the most suitable model for disease severity diagnosis.

### 3.1 Dataset

**FIGURE 1:**
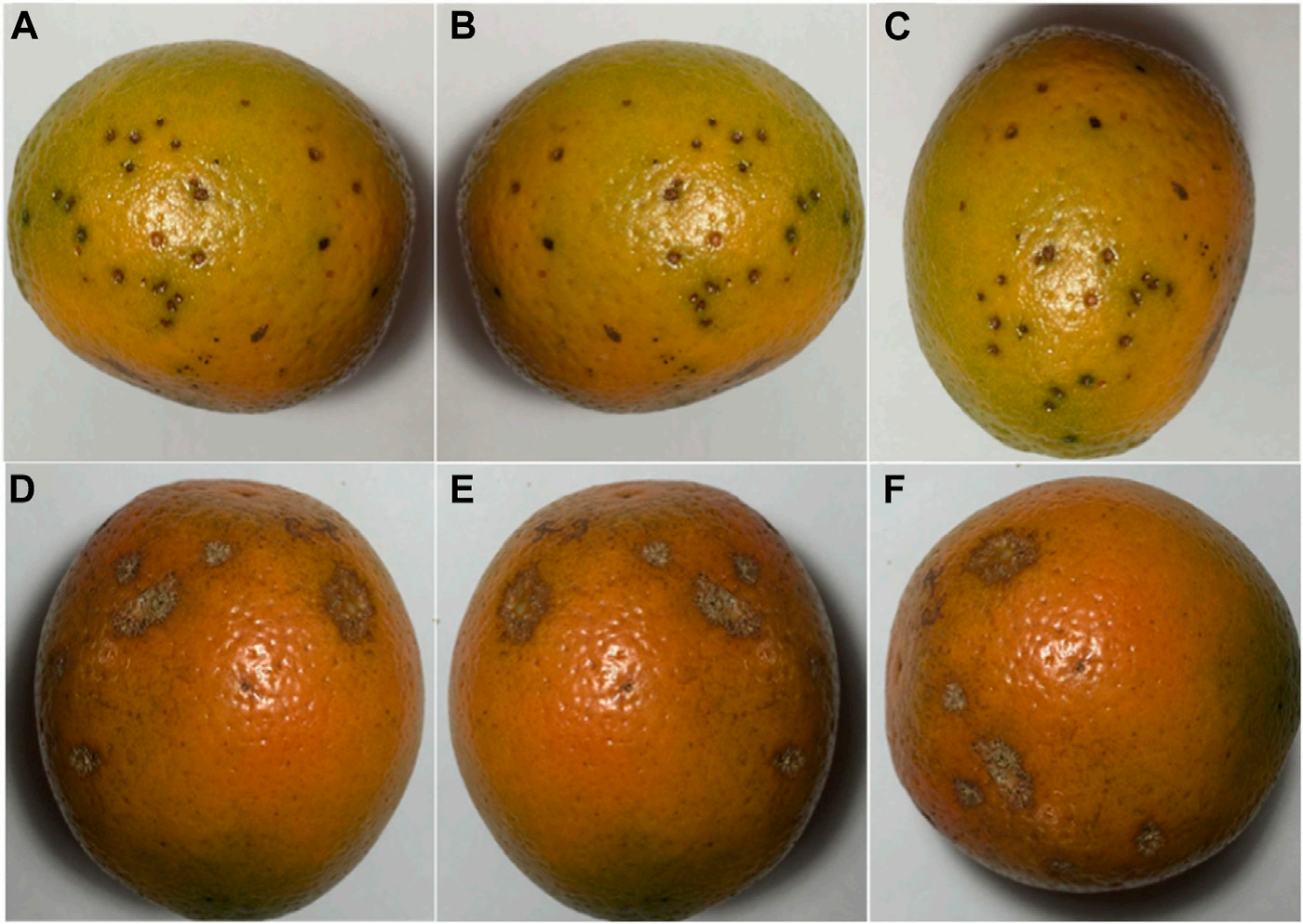
Citrus dataset: **(A)** Citrus black spot; **(B)** Citrus black spot after mirror flip; **(C)** Citrus black spot after rotation; **(D)** Citrus canker; **(E)** Citrus canker after mirror flip; **(F)** Citrus canker after rotation.

The dataset for this article is mainly from the Kaggle website, which includes 800 images for the citrus fruit black spot and canker diseases. All of them are taken in a uniform laboratory setting, and some sample images are shown in Figure 1.

For effective differentiation in the experiment, fresh citrus images without disease are added for classification based on these two disease images. In addition to this, the disease images are expanded to 2,000 by data enhancement operations, including mirror flip and angular rotation. Moreover, of these 2,000 images, the number of black spots and cankers each accounted for 50%. At the stage of severity diagnosis, Manual pixel-level labels of the raw dataset are made by using an annotation tool named labelme (Marois and Syssau, 2008), image pixels are labelled in one of four categories: healthy, black spotted, cankered and background. We use different colors to differentiate.

### 3.2 EfficientNetV2

EfficientNet is considered the best CNN network when it is first proposed. It improves the performance of the network by simultaneously improving the width, depth and resolution of the network. However, with the improved performance, EfficientNet also has its drawbacks: 1) The training period is limited by the size of the input image and becomes extremely inefficient when the image size is too large. 2) Premature use of deep convolution can make the model counterproductive. 3) Equivalent amplification of each stage is suboptimal. These drawbacks limited EfficientNet and prevented it from being widely used in practice until the advent of EfficientNetV2.

**FIGURE 2:**
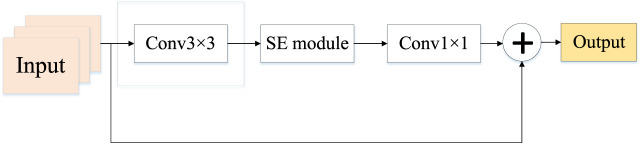
Structure of Fused-MBConv module.

EfficientNetV2 replaces the originally available MBConv by Fused-MBConv based on EfficientNet and proposes an improved progressive learning method that can not only improve the training speed but also the accuracy rate. Fused-MBConv replaces expansion conv1x1 and depth-wise conv3x3 in the main branch with a normal conv3x3, as shown in Figure 2. However, structure replacement like this does not happen at every layer, if the shallow MBConv structure is replaced with a Fused-MBConv structure, the training speed can be significantly improved, but if one is to use all Fused-MBConv modules instead, the training period would rise significantly with the increase in computational complexity. So the best combination of MBConv and Fused-MBConv is searched in EfficientNetV2 using NAS technology.

**FIGURE 3:**
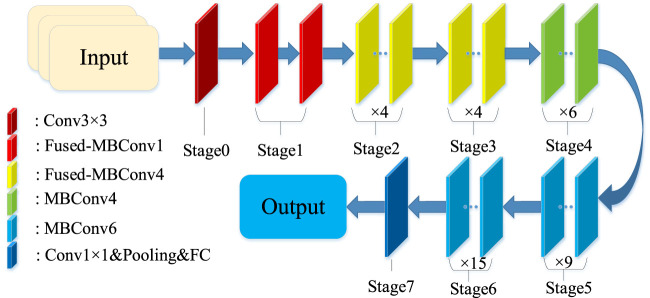
Structure of EfficientNetV2-S.

Similar to EfficientNet, which includes several models from B0-B7, EfficientNetV2 also includes several classical models, namely, EfficientNetV2-S, EfficientNetV2-M and EfficientNetV2-L. In our experiments, EfficientNetV2-S is used as a base model. The structure of the EfficientNetV2-S is shown in Figure 3.

### 3.3 Transfer learning

The advent of TL has brought new life to DL models, where weights trained on the initial training set are migrated to the target network to reduce training cycles and improve model accuracy. Using TL means that instead of training with randomly initialized weights from start to finish, weights for up-training on some large labelled datasets (e.g., public image datasets, etc.) can be obtained by pre-training them and using them as a way to initialize the target network weights. In this paper, pre-trained models learned from ImageNet are considered and transferred to the target dataset for task-specific training. Pre-training weights trained on ImageNet are imported into the EfficientNetV2 model as a way to improve the classification performance of the model.

### 3.4 Proposed approach

As mentioned in Section 3.2, EfficientNet represents the most advanced CNN model framework. It further reduces the computational complexity and improves performance. However, EfficientNetV2’s thin final stage layer caught our attention. The gap before pooling and 1 × 1 convolution mean that the final layer of the EfficientNetV2 model does not allow for the extraction of multi-scale features, which will inevitably have an impact on the final classification. The first few convolutional layers of a convolutional neural network are usually used to extract colour and corner point features of an image, while the end layer performs the resolution of weights and computation of features, so the lack of performance of the end layer is critical to the overall network model. In addition, the MBConv in EfficientNetV2 also inspires and reminds us of the Inception module (de Melo et al., 2022), which is similar to it. The Inception module is added to the final phase of EfficientNetV2 to enhance network performance.

**FIGURE 4:**
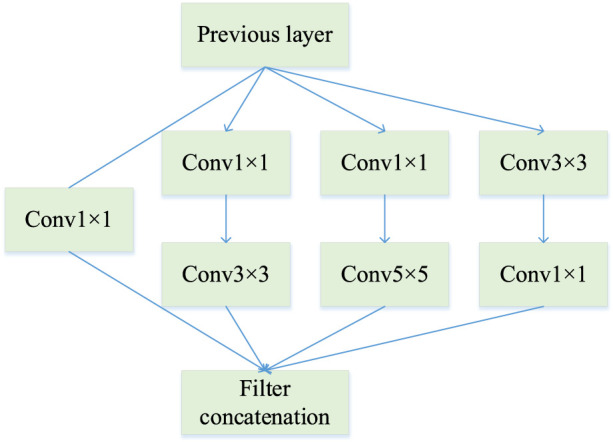
Structure of InceptionV1.

MBConv is an inverted linear bottleneck layer with depth-separated convolution, Inception is a module that is a discrete spectrum between normal convolution and convolution along depth-separated convolution, compared to normal convolution, the major difference between these two types of convolutions is the much-reduced number of parameters. Suppose the input feature map dimension is $\left(H_{1}\times W_{1}\times M\right)$ , where $H_{1}$ , $W_{1}$ , and M is the height, width and number of channels of the input feature map respectively. The convolution kernel size is $\left(D_{K}\times D_{K}\right)$ , where $D_{K}$ is the height and width of the convolution kernel. The output feature map dimension is $\left(H_{O}\times W_{O}\times N\right)$ , where $H_{O}$ , $W_{O}$ , and N is the height, width and number of channels of the output feature map respectively. For the standard convolution, the computational complexity can be calculated as

$$F_{1}=H_{O}\times W_{O}{\times D}_{K}\times D_{K}\times N\times M,$$ (1)

where $F_{1}$ is the computation of the standard convolution. Depth-separable convolution can be calculated as

$$F_{2}=H_{O}\times W_{O}{\times D}_{K}\times D_{K}\times M+H_{O}\times W_{O}\times N\times M,$$ (2)

where $F_{2}$ is the computation of the depth-separable convolution. The ratio between the two calculations can be found as

$$R=\frac{F_{2}}{F_{1}}=\frac{1}{N}+\frac{1}{D_{K}\times D_{K}},$$ (3)

where R is the ratio of the calculation volumes of the two calculation methods. The advantage of the Inception module is that it allows the aggregation of visual information of different sizes, while first down-scaling larger matrices to facilitate feature extraction from different scales. The framework of the InceptionV1 module used in this paper is shown in Figure 4, which replaces vertically stacked convolutions with parallel convolutions. This module uses three different scales of convolution kernels $\left(1\times 1,3\times 3,5\times 5\right)$ and a maximum pooling kernel $\left(3\times 3\right)$ to increase the adaptability of the network to different scales.

Features from the previous layer are extracted and stitched together at the end after passing through these three different sizes of convolution kernels. This means that the network can perceive local areas of the image from different sizes in the same layer and fuse features from different scales. Thus, InceptionV1 has the following advantages over standard convolution:

- Control the computational complexity while increasing the parameters.
- The multi-scale processing performance of the network is improved by aggregating feature information.

In this paper, a 2-layer InceptionV1 module is added to the final stage between the 1 × 1 convolutional layer and the global pooling layer, moreover, the newly generated network architecture is shown in Table 1.

**TABLE 1:** Parameters related to the proposed network model.

Stage	Type	Kernel size	Stride	Layers
0	Conv3 × 3	3 × 3	2	1
1	Fused-MBConv1	3 × 3	1	2
2	Fused-MBConv4	3 × 3	2	4
3	Fused-MBConv4	3 × 3	2	4
4	MBConv4	3 × 3	2	6
5	MBConv6	3 × 3	1	9
6	MBConv6	3 × 3	2	15
7	InceptionV1	-	1	2
	Conv1 × 1&Pooling&FC	-	-	1

The excellent multi-scale inference capability of the InceptionV1 model fills in well the lack of feature extraction capability before the descending convolution of the tail layer. It both picks up the feature extraction from the previous stage and prepares the ground for the dimensionality reduction operation in the next part. Therefore, the newly generated network usually consists of two parts: the first part is the pre-training module, stages 0–6, which is used for basic feature extraction; the second part is the extension layer, stage 7, which is used for extracting high-latitude features and using multi-scale feature maps for classification. In addition, the training of the model is performed using two-TL with the following training strategy. In the first step, model parameters are inferred from scratch while freezing the weights from the bottom multiscale module (stage 7) pre-trained from ImageNet. The second step retrains all weights by loading the model imported in the first stage of training and using the target citrus dataset. The multiscale module at the bottom of the model has initial weights and is trained using the citrus dataset, thus network performance is improved.

### 3.5 Severity diagnosis

Severity diagnosis is one of the sub-tasks of semantic segmentation, which aims to calculate the severity of disease by accurately measuring the area of the diseased region, calculated as

$$S=\frac{DA}{TA},$$ (4)

where S is the severity of the disease, DA is the area of the disease area and TA is the total fruit area.

**FIGURE 5:**
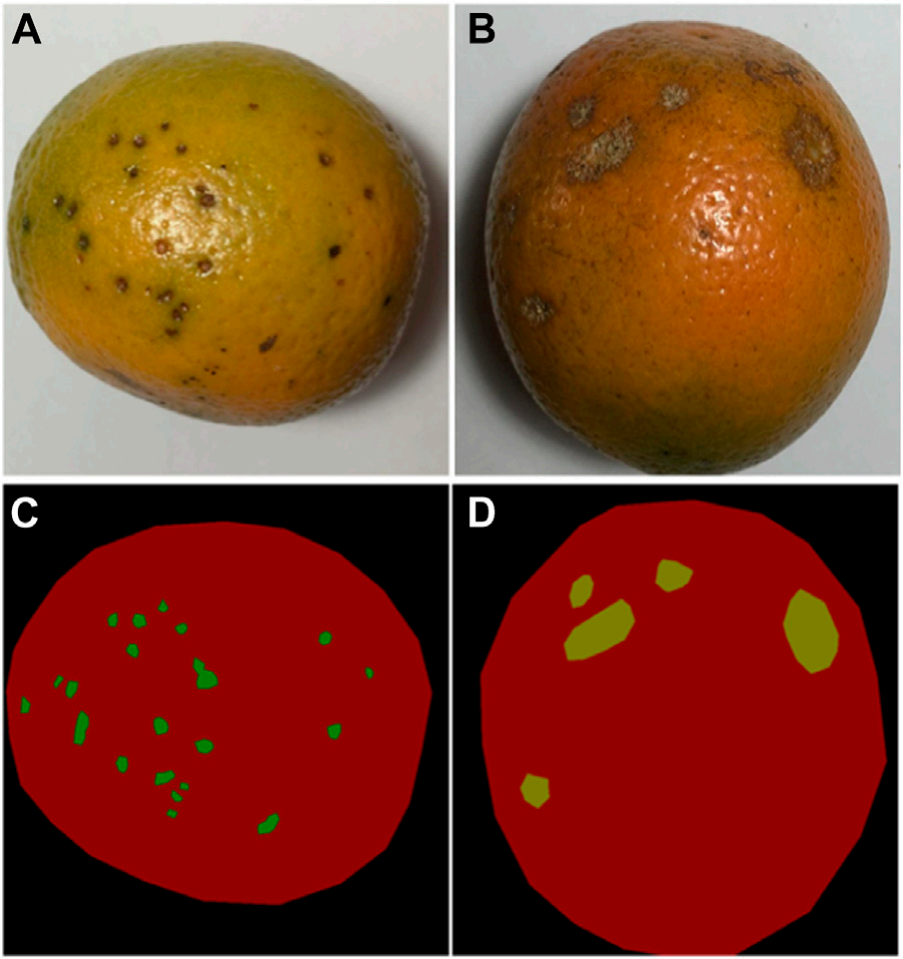
Images of citrus fruit before and after labelling: **(A)** Original black spot image; **(B)** Original canker disease image; **(C)** Image of the black spot after labelling; **(D)** Image of canker disease after labelling.

Using the label annotation of the dataset in Section 3.1, 400 images are obtained for the two citrus fruit diseases shown in Figure 5. Each disease category dataset is divided into subsets for training, validation and testing in proportions of 70%, 10% and 20% respectively.

**FIGURE 6:**
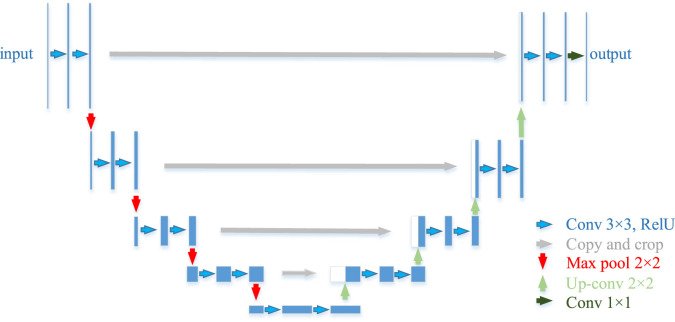
VGG-U-Net Architecture.

The U-Net, as the name suggests, is a U-shaped network architecture, divided into a down-sampling part (the backbone feature extraction network) and an up-sampling part (the enhanced feature extraction network). The “U” structure of its features consists of conventional convolution and maximum pooling forming the down-sampling, followed by a mirroring up-sampling step. In this work, the down-sampling part of U-Net will be replaced by VGG16 to enhance feature extraction from its backbone feature network. 3 × 3 convolution is used in the same horizontal layer as the ReLu activation function and is carried through to the next dimension by 2 × 2 maximum pooling. The last step of each horizontal layer is connected to its associated up-sampling block in the upstream path as shown in Figure 6. Similarly, in the training phase, the VGG16 weights pre-trained on ImageNet are imported into the model with the help of TL to shorten its training cycle. The U-Net model is then retrained on the citrus fruit dataset and the parameters are fine-tuned to obtain the optimal weights. Finally, the test output of the model is compared with the real labels and its performance is analyzed.

## 4 Results

This section provides the results of the qualitative analysis of the disease detection models presented and the quantitative analysis of the severity diagnosis models in Section 3, which analyses the performance of these two types of models in detail.

### 4.1 Experimental configuration and parameters

In this paper, experiments are conducted using the Python3 programming language, and the models are implemented using the Tensorflow 2.0 (Mart´ın et al., 2016) framework. In addition, the training and testing of network models in this paper are performed on an AMD5700G and an NVIDIA A6000. In the model training stage, set the training batch size to 8, and a stochastic gradient descent algorithm is chosen to optimize the parameters. The initial learning rate is set to 0.01 and decreased with training epochs. Meanwhile, the momentum is set to 0.9 for accelerating convergence. The dropout is set to 0.1 for preventing overfitting.

### 4.2 Disease recognition results

Based on the model proposed in Section 3.4, this section trained and tested the model on the citrus fruit dataset. To fairly evaluate the performance of the model, each class of citrus fruit is randomly and evenly divided into 5 portions. Four of these are used to train the fine-tuned model and the remaining one is used to test and evaluate the model’s performance. In addition, we use K-fold cross-validation (K = 5) for model training and hyperparameter selection. Thus the ratio of the training, validation and test sets for the experiment is set to 6:2:2. Five different fine-tuned models are obtained after cross-validation. Where the accuracy of a single model is calculated by

$$A=\frac{1}{n}\left\{\begin{array}{c} 1,f\left(x_{i}\right)=y_{i}\\ 0,f\left(x_{i}\right)!=y_{i} \end{array}\right.,$$ (5)

where A is the accuracy, n is the number of inputs, $f\left(x_{i}\right)$ is the predicted outcome of the model and $y_{i}$ is its true label. These 5 model results are combined to obtain the average accuracy of the model. The average accuracy avoids the errors caused by single training and gives us a more objective view of the model’s performance.

**FIGURE 7:**
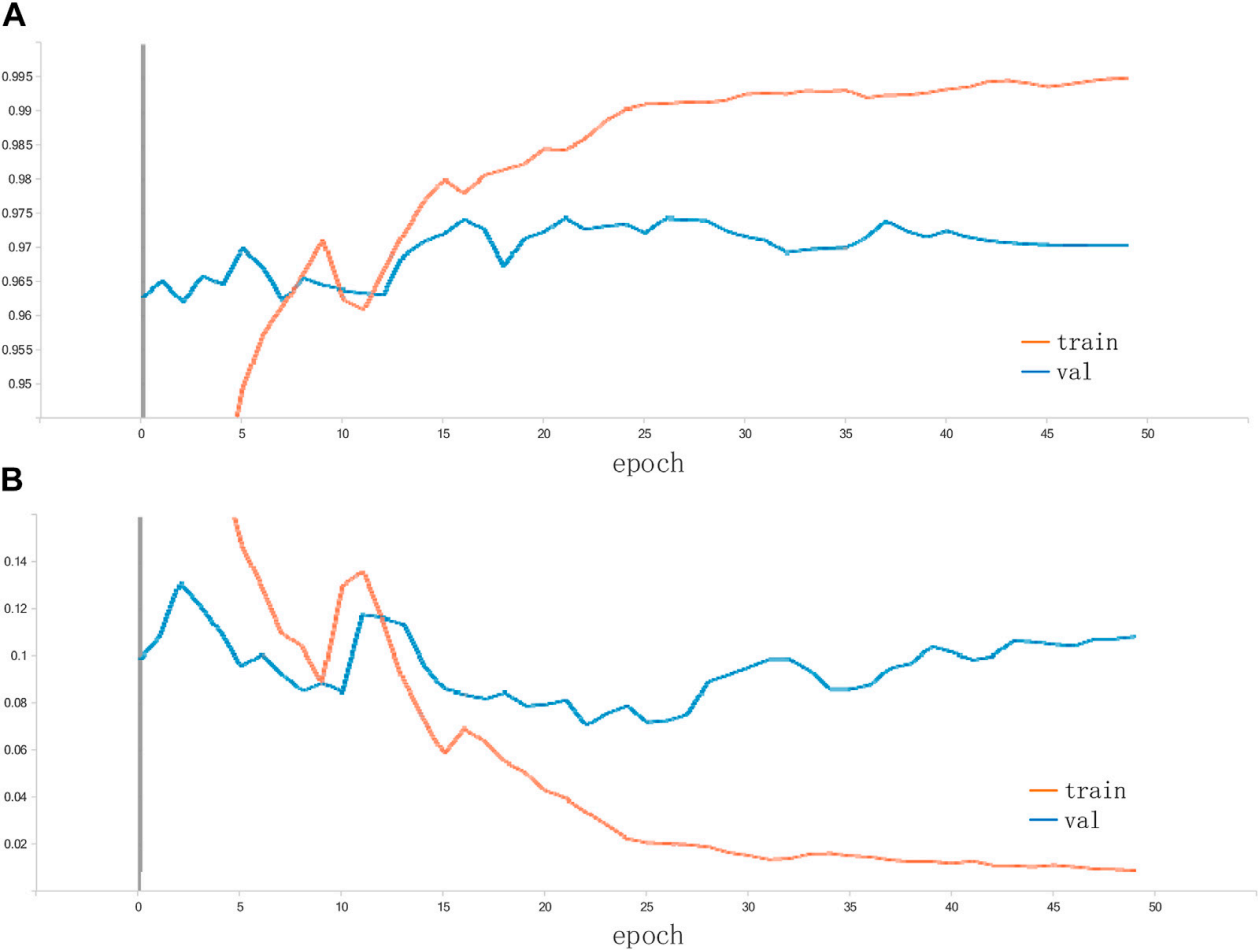
Accuracy and loss of the proposed method on the citrus dataset: **(A)** accuracy; **(B)** loss.

Table 2 shows the test results of the 5 training sessions and what can be seen is that although there is some fluctuation in the results of the 5 data sessions, the overall performance is at a high level. In addition, to further assess the feasibility of the proposed methodology, three classical and convincing CNN models are added to the comparison experiments, including ResNet50, GoogleNet, and EfficientNet. Again, these network models are loaded with weights pre-trained on ImageNet. Again, these network models are loaded with weights pre-trained on ImageNet. The TL strategy similar to the proposed method is used to shorten the training cycle, training is performed on randomly assigned image data to compare the performance strengths and weaknesses of each network. The accuracy of the proposed method on the citrus fruit dataset is shown in Figure 7. The proposed model performs extremely well on the citrus fruit dataset, as can be seen from the figure, the loss function and accuracy converge at 25 training sessions, one of the surprising things is that the accuracy is above 99% and the losses converge to 0%. We have repeated the experiments many times by using different epochs (including epoch = 30, 40 and 50). The results show that the model’s performance has reached convergence at epoch = 25, and the accuracy of the subsequent rounds always fluctuates, so this paper concludes that the model can obtain good performance at epoch = 25. Table 3 shows the average accuracy of each model after 5 training experiments.

**TABLE 2:** Results for 5 training sessions of the model.

-	1st fold	2nd fold	3rd fold	4th fold	5th fold	Average
Accuracy	97.5	98.4	98.7	97.2	99.2	98.2
Loss	0.123	0.076	0.076	0.112	0.043	0.086

**TABLE 3:** Performance comparisons with other approaches.

Pre-trained model	Average accuracy (train)%	Average accuracy (val)%	Average accuracy (test)%	Average loss	Average time (each epoch)
ResNet50 Ji and Wu, (2022)	96.6	94.9	90.2	0.123	17′25″
GoogleNet qun PAN et al., (2022)	97.7	96.4	92.5	0.100	2′05″
EfficientNet Espejo-Garcia et al., (2022)	96.0	94.3	89.6	0.335	23′43″
EfficientNetV2 Tan and Le, (2021)	97.3	95.2	92.9	0.194	32′38″
EfficientNetV2 Tan and Le, (2021) (with TL)	97.7	95.3	92.9	0.180	9′12″
Proposed method (without TL)	98.6	97.8	95.2	0.106	34′47″
Proposed method	99.3	98.2	95.6	0.086	9′22″

**FIGURE 8:**
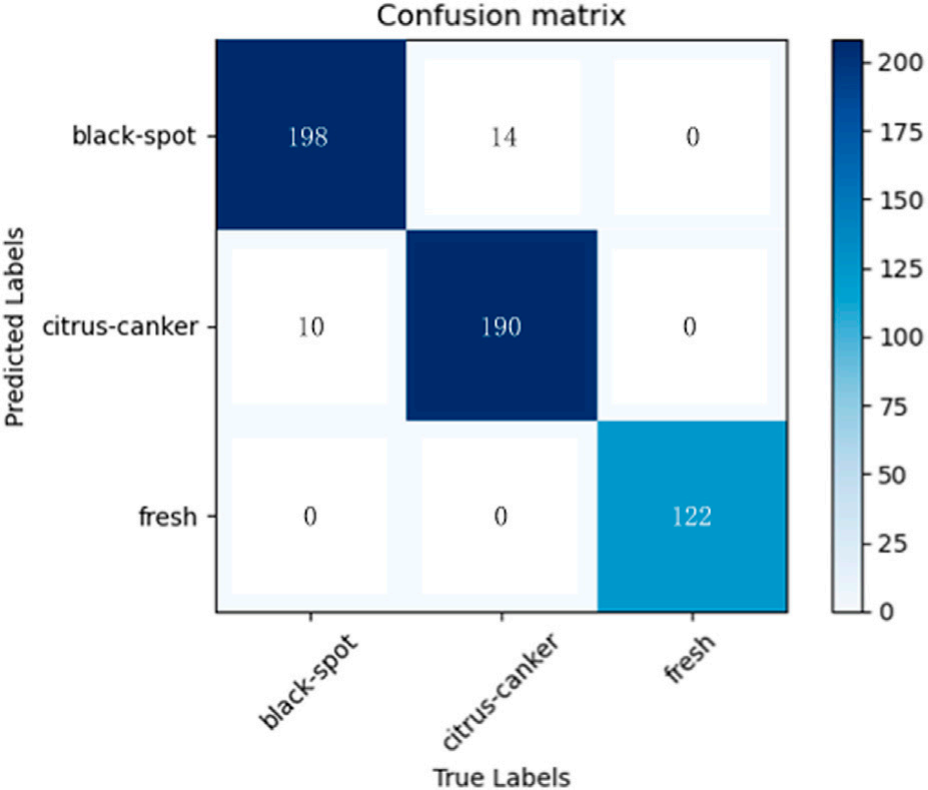
Confusion matrix for the proposed method.

It can be seen that the proposed model outperforms the comparison methods on the citrus fruit dataset. In particular, despite the increase in training time compared to the baseline EfficientNetV2. However, it shows a large improvement in accuracy and a decrease in training loss compared to the EfficientNetV2. This means that the combination of Inception module and EfficientNetV2 can enhance the feature extraction ability of EfficientNetV2 to some extent and improve the performance of the classifier. The main reason for this is that all other networks are single-layer networks with only a classification layer, while the proposed model applies the Inception module to the tail-layer classification, extracting high latitude for the final classification. In addition, the use of TL significantly reduces the training cycle of the model. The training time is reduced to 1/3 results in faster convergence and improved performance. The reason is that in transfer learning the weights of the first few layers of the model is froze and the pre-trained parameters are imported. This eliminates the need to train the model from scratch and greatly reduces the training period. In summary, the proposed model combines the advantages of EfficientNetV2 and the Inception module, including the former excellent basic feature extraction ability and the latter excellent multi-scale feature extraction ability, which results in such a perfect performance on the citrus fruit dataset. After five experiments with 50 epochs of training each, the average test accuracy of 95.6% and the average loss of 0.01 are obtained. The results of the five predictions made on the test set are averaged and Figure 8 shows the average confusion matrix of the test results. It can be seen that all the test samples were well classified. This shows that the proposed model is effective in identifying citrus fruit diseases.

### 4.3 Disease severity diagnosis

To verify the effectiveness of the VGG-U-Net segmentation model, this paper conducts segmentation experiments on the DeeplabV3, U-Net and VGG-U-Net models respectively under the same segmentation dataset, the hyper-parameters of the experiments are uniformly set to an initial learning rate of 0.0001, the training number is 100 rounds. If the loss of validation does not decrease, training will end early to prevent it from being over-fitted. In addition, to show the segmentation performance of the model more intuitively, the Mean Intersection Ratio (MIoU), Mean Pixel Accuracy (MPA) and Precision are used to evaluate the segmentation performance of the model species. The more these indicators converge to 1, the better the segmentation performance. The calculation process for the evaluation indicators is described as follows:

$$MIoU=\frac{1}{k+1}\sum_{i=0}^{k}\frac{TP}{FN+FP+TP},$$ (6)

$$MPA=\frac{1}{k+1}\sum_{i=0}^{k}\frac{TP+TN}{FN+FP+TP+TN},$$ (7)

$$P=\frac{TP}{TP+FP},$$ (8)

where k is the number of validations, TP (true positive) means the label is true and the prediction is true, FN (false negative) means the label is false and the prediction is true, FP (false positive) means the label is true and the prediction is false and TN (true negative) means the label is false and the prediction is false, P is the Precision.

The test results for all detection models are shown in Table 4. Among them, VGG-U-Net shows the best performance, achieving an average pixel accuracy of 87.66%, which is better than the base U-Net model and has a large performance improvement compared to DeeplabV3. Therefore, building on U-Net is the right choice, using VGG to replace the U-Net coding backbone extraction network can enhance the feature extraction capability of U-Net, which is more conducive to the segmentation in the decoding stage and improve the segmentation performance of the network.

**TABLE 4:** Detection results of the three segmentation models.

Model	Metrics	Category				Average value (%)
		Canker	Black-spot	Orange	Background	
DeeplabV3 Wang and Liu, (2021)	IoU	0.56	0.28	0.95	0.97	69.01
	PA	0.60	0.30	0.99	0.98	71.46
	Precision	0.91	0.83	0.96	0.99	92.31
U-Net de Melo et al., (2022)	IoU	0.79	0.51	0.97	0.97	80.85
	PA	0.86	0.63	0.99	0.99	86.65
	Precision	0.90	0.72	0.98	0.99	89.66
VGG-U-Net Wspanialy and Moussa, (2020)	IoU	0.80	0.56	0.97	0.97	82.53
	PA	0.89	0.65	0.98	0.99	87.66
	Precision	0.89	0.80	0.98	0.99	91.56

It is worth noting that the segmentation performance of the three segmentation models for the black spot is much less than that of canker disease, due to the small size and often scattered distribution of black spots, which greatly increased the difficulty of detecting segmentation, whereas the large area and dense distribution of canker disease greatly helped the performance of the segmentation models.

## 5 Conclusion

In this paper, a citrus disease detection system is proposed, which has the function of diagnosing and detecting the severity of citrus diseases. The disease identification phase focuses on the model’s high-latitude feature extraction capabilities. The proposed model revolves around the extraction of multi-scale information from images, which combines the state-of-the-art EfficientNetV2 with the classical Inception module. In addition, TL methods are applied to the model to reduce training cycles and improve accuracy. Experiment results show that the proposed model has produced the best performance compared to the classical CNN. In the disease severity diagnosis stage of the fruit, three different segmentation models are compared, and their performance in terms of pixel-level accuracy is evaluated. Results show that the VGG-U-Net has the highest average accuracy, proving the effectiveness of replacing the underlying U-Net with VGG to encode the backbone feature extraction network. Future work will enhance the performance of the segmentation network for the detection of small spot targets and extend this system to other crops.

## Data Availability

Publicly available datasets were analyzed in this study. This data can be found here: https://www.kaggle.com/datasets/jonathansilva2020/orange-diseases-dataset.

## References

[B1] Abdelsalam A. M. Sayed M. S. (2016). “Real-time defects detection system for orange citrus fruits using multi-spectral imaging,” in Midwest symposium on circuits and systems, 1–4.

[B2] Avery K. R. Pan J. Engler-Pinto C. C. Wei Z. Yang F. Lin S. (2014). Xception: deep learning with depthwise separable convolutions. SAE Int. J. Mat. Manuf. 7 (3), 560–566. 10.4271/2014-01-0975

[B3] Behera S. K. Jena L. Rath A. K. Sethy P. K. (2018). “Disease classification and grading of orange using machine learning and fuzzy logic,” in Proceeding of the International Conference on Communication and Signal Processing (ICCSP), April 2018, Chennai, India, IEEE, 0678–0682.

[B4] Chao X. Hu X. Feng J. Zhang Z. Wang M. He D. (2021). Construction of apple leaf diseases identification networks based on Xception fused by SE module. Appl. Sci. 11 (10), 4614–4628. 10.3390/app11104614

[B5] Chen Y. Wu J. Cui M. (2018). “Automatic classification and detection of oranges based on computer vision,” in Proceeding of the IEEE 4th International Conference on Computer and Communications, ICCC, December 2018, Chengdu, China, IEEE, 1551–1556.

[B6] Cheng M. Galimzianova A. Lesjak Ž. Špiclin Ž. Lock C. B. Rubin D. L. (2018). Deep learning in medical image analysis and multimodal learning for clinical decision support, 11045.

[B7] Conti G. Gardella V. Vandecaveye M. A. Gomez C. A. Joris G. Hauteville C. (2020). Transgenic citrange troyer rootstocks overexpressing antimicrobial potato snakin-1 show reduced citrus canker disease symptoms. J. Biotechnol. 324, 99–102. 10.1016/j.jbiotec.2020.09.010 32998033

[B8] da Costa A. Z. Figueroa H. E. H. Fracarolli J. A. (2020). Computer vision based detection of external defects on tomatoes using deep learning. Biosyst. Eng. 190 (25), 131–144. 10.1016/j.biosystemseng.2019.12.003

[B9] de Melo M. J. Gonçalves D. N. Gomes M. d. N. B. Faria G. Silva J. d. A. Ramos A. P. M. (2022). Automatic segmentation of cattle rib-eye area in ultrasound images using the UNet++ deep neural network. Comput. Electron. Agric. 195, 106818. 10.1016/j.compag.2022.106818

[B10] Deng L. Li J. Han Z. (2021). Online defect detection and automatic grading of carrots using computer vision combined with deep learning methods. LWT 149 (2020), 111832. 10.1016/j.lwt.2021.111832

[B11] Espejo-Garcia B. Malounas I. Mylonas N. Kasimati A. Fountas S. (2022). Using EfficientNet and transfer learning for image-based diagnosis of nutrient deficiencies. Comput. Electron. Agric. 196, 106868. 10.1016/j.compag.2022.106868

[B12] Fan S. Li J. Zhang Y. Tian X. Wang Q. He X. (2020). On line detection of defective apples using computer vision system combined with deep learning methods. J. Food Eng. 286 (2019), 110102. 10.1016/j.jfoodeng.2020.110102

[B13] Hassan S. M. Maji A. K. Jasiński M. Leonowicz Z. Jasińska E. (2021). Identification of plant-leaf diseases using CNN and transfer-learning approach. Electron 10 (12), 1388–1407. 10.3390/electronics10121388

[B14] Iandola F. N. Han S. Moskewicz M. W. Ashraf K. Dally W. J. Keutzer K. (2016). SqueezeNet: AlexNet-level accuracy with 50x fewer parameters and <0.5MB model size. arXiv Prepr. arXiv1602.07360, 1–13.

[B15] Ismail N. Malik O. A. (2021). Real-time visual inspection system for grading fruits using computer vision and deep learning techniques. Inf. Process. Agric. 110 (43), 1–14. 10.1016/j.inpa.2021.01.005

[B16] Ji M. Wu Z. (2022). Automatic detection and severity analysis of grape black measles disease based on deep learning and fuzzy logic. Comput. Electron. Agric. 193 (24), 106718. 10.1016/j.compag.2022.106718

[B17] Khan A. I. Quadri S. M. K. Banday S. Latief Shah J. (2022). Deep diagnosis: A real-time apple leaf disease detection system based on deep learning. Comput. Electron. Agric. 198, 107093. 10.1016/j.compag.2022.107093

[B18] Lee S. H. Chan C. S. Mayo S. J. Remagnino P. (2017). How deep learning extracts and learns leaf features for plant classification. Pattern Recognit. 71 (1), 1–13. 10.1016/j.patcog.2017.05.015

[B19] Marois B. Syssau P. (2008). LabelMe: A database and web-based tool for image annotation. Int. J. Comput. Vis. 32 (1–3), 157–173. 10.1007/s11263-007-0090-8

[B20] Mart´ın A. Chen Z. Paul B. Chen J. (2016). “{TensorFlow}: A system for {Large-Scale} machine learning,” in Proceeding of the 12th USENIX symposium on operating systems design and implementation (OSDI 16), November 2–4, 2016, Savannah, GA, USA, 265–283.

[B21] qun Pan S. Qiao J. f. Wang R. Yu H. l. Wang C. Taylor K. (2022). Intelligent diagnosis of northern corn leaf blight with deep learning model. J. Integr. Agric. 21 (4), 1094–1105. 10.1016/s2095-3119(21)63707-3

[B22] Raikar M. M. Meena S. M. Kuchanur C. Girraddi S. Benagi P. (2020). Classification and grading of okra-ladies finger using deep learning. Procedia Comput. Sci. 171 (2019), 2380–2389. 10.1016/j.procs.2020.04.258

[B23] Sabzi S. Abbaspour-Gilandeh Y. Arribas J. I. (2017). “Non-intrusive image processing thompson orange grading methods,” in Proceeding of the 56th FITCE Congress, September 2017, Madrid, Spain, IEEE, 35–39.

[B24] Simonyan K. Zisserman A. (2014). “Very deep convolutional networks for large-scale image recognition,” in 3rd int. Conf. Learn. Represent. ICLR 2015 - conf. Track proc., 1–14.

[B25] Szegedy C. Liu W. Jia Y. Sermanet P. Reed S. Angeulov D. (2015). “Going deeper with convolutions,” in Proceeding of the IEEE Conference on Computer Vision and Pattern Recognition (CVPR), IEEE, 1–9.

[B26] Tan M. Le Q. V. (2019). “EfficientNet: rethinking model scaling for convolutional neural networks,” in 36th international conference on machine learning, ICML, 6105–6114.

[B27] Tan M. Le Q. V. (2021). “EfficientNetV2: smaller models and faster training,” in Proceedings of the 38th international conference on machine learning, 10096–10106.

[B28] Vasconez J. P. Delpiano J. Vougioukas S. Auat Cheein F. (2020). Comparison of convolutional neural networks in fruit detection and counting: A comprehensive evaluation. Comput. Electron. Agric. 173 (2019), 105348. 10.1016/j.compag.2020.105348

[B29] Wang J. Liu X. (2021). Medical image recognition and segmentation of pathological slices of gastric cancer based on Deeplab v3+ neural network. Comput. Methods Programs Biomed. 207 (1), 106210. 10.1016/j.cmpb.2021.106210 34130088

[B30] Wang G. Sun Y. Wang J. (2017). Automatic image-based plant disease severity estimation using deep learning. Comput. Intell. Neurosci. 2017 (1), 2917536–2917538. 10.1155/2017/2917536 28757863 PMC5516765

[B31] Wspanialy P. Moussa M. (2020). A detection and severity estimation system for generic diseases of tomato greenhouse plants. Comput. Electron. Agric. 178, 105701. 10.1016/j.compag.2020.105701

[B32] Wu Z. Shen C. van den Hengel A. (2019). Wider or deeper: revisiting the ResNet model for visual recognition. Pattern Recognit. 90, 119–133. 10.1016/j.patcog.2019.01.006

[B33] Yang Z. Zhang L. Zhao J. f. Zhang X. k. Wang Y. Li T. s. (2022). New geographic distribution and molecular diversity of citrus chlorotic dwarf-associated virus in China. J. Integr. Agric. 21 (1), 293–298. 10.1016/s2095-3119(20)63601-2

[B34] Zhou C. (2020). The status of citrus huanglongbing in China. Trop. Plant Pathol. 45 (3), 279–284. 10.1007/s40858-020-00363-8

